# Dynamic economic-entropy regulation of community-scale green hydrogen supply chains with carbon-microgrid coupling

**DOI:** 10.1016/j.isci.2026.115504

**Published:** 2026-03-26

**Authors:** Guohui Lan, Yashu Chen, Chunzhong Li, Jianming Wang

**Affiliations:** 1 School of Economics and Management, Anhui University of Science and Technology, Huainan, Anhui 232001, China; 2 School of Statistics and Applied Mathematics, Anhui University of Finance and Economics, Bengbu, Anhui 233030, China

**Keywords:** Applied sciences, Electrical engineering, Energy sustainability

## Abstract

Deep decarbonization through green hydrogen deployment faces economic uncertainty and market coordination challenges. This study develops a dynamic economic entropy regulation framework that transforms uncertainty from a passive diagnostic attribute into an actively controllable system variable. By embedding entropy minimization within a deep reinforcement learning-based closed-loop optimization architecture, proactive uncertainty regulation is achieved across the green hydrogen supply chain. A ternary coupling model integrating carbon trading, green hydrogen systems, and community-scale smart microgrids quantifies carbon price transmission effects. Analysis of 10-year empirical data across five community archetypes demonstrates 55.4% reduction in system-level economic entropy, over 90% renewable energy utilization, and enhanced investment performance under realistic carbon price regimes. Life cycle assessment shows 81.7% lower global warming potential compared with gray hydrogen production.

## Highlights

• Economic entropy reconceptualized as dynamic control objective for uncertainty regulation
• Ternary carbon-hydrogen-electricity coupling reveals carbon pricing transmission mechanisms
• Deep reinforcement learning achieves 55.4% entropy reduction and 90%+ renewable utilization
• Life cycle assessment shows 81.7% lower global warming potential versus gray hydrogen

## Graphical abstract

**Figure:**
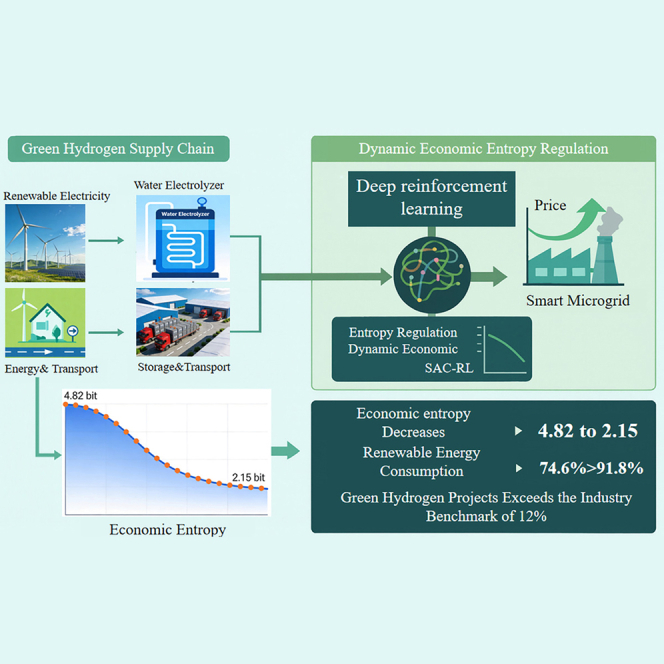

## Introduction

The global energy system is undergoing a profound structural transformation driven by the urgent need to mitigate climate change and achieve long-term carbon neutrality. Within this transition, green hydrogen has emerged as a critical zero-carbon energy carrier capable of decarbonizing hard-to-abate sectors and enabling deep integration of renewable energy sources. Despite its strategic importance, large-scale commercialization remains highly uncertain. Across the entire supply chain—spanning renewable electricity generation, hydrogen production, storage, transportation, and end-use consumption—projects are exposed to compounded economic risks arising from renewable intermittency, spatiotemporal demand fluctuations, and volatile carbon market signals.^1^^,^^2^ These interacting uncertainties have resulted in persistently high investment risks, substantially constraining deployment pace and scale.

Community-scale smart microgrids are increasingly being recognized as pivotal infrastructures for integrating distributed renewable energy and enhancing system flexibility.^3^ Their coupling with green hydrogen systems offers significant potential to improve energy utilization efficiency, provide long-duration storage, and enhance resilience under extreme conditions. However, existing research lacks a unified theoretical framework capable of systematically integrating green hydrogen supply chains, carbon trading mechanisms, and smart microgrids within a coherent uncertainty management paradigm. Consequently, current planning and operational strategies remain fragmented and reactive.

Recent empirical studies underscore this challenge. Odenweller et al. reported that only approximately 7% of globally announced green hydrogen capacity had been realized, as planned by 2023, highlighting the disconnect between policy ambition and operational outcomes.^4^ This gap reflects systemic uncertainty and coordination failures across markets and actors. Economic entropy theory has gained traction as a quantitative tool for characterizing market complexity,^5^^,^^6^ yet applications remain fundamentally static and post-hoc, providing diagnostic insight without mechanisms for real-time uncertainty control.

Prior studies have addressed specific subsystems but not their integration. Zhou et al. proposed a coupling mechanism that integrates green certificate trading with stepped carbon trading.^7^ Han et al. demonstrated that hydrogen storage enhances distribution network resilience under N-m fault scenarios (where N represents the total number of system components and m denotes the number of failed components).^8^ Li et al. introduced distributionally robust optimization for renewable energy and electric vehicle uncertainty.^9^ However, these methods could neither achieve simultaneous integration of carbon trading, green hydrogen, and smart microgrids nor address uncertainty propagation across these coupled domains. Entropy-based methods in power grids^10^ and behavioral economics^11^ similarly lack feedback mechanisms for operational control.^12^

Green hydrogen deployment is fundamentally constrained by economic competitiveness. The levelized cost of hydrogen (LCOH) currently ranges 3–6 USD/kg, insufficient for sustained competitiveness with fossil-based hydrogen.^13^ Electrolyzer costs, dominant contributors to LCOH, may decline from 500 to 1400 USD/kW to below 500 USD/kW by 2030, with renewable cost reductions expected to lower LCOH to 2.0–2.5 USD/kg by 2035.^14^ Policy instruments—particularly carbon pricing—improve competitiveness, especially under regimes like the EU Carbon Border Adjustment Mechanism.^15^^,^^16^ However, most analyses assume stable markets, offering limited insight into dynamic uncertainty propagation.

Economic entropy theory provides a mathematically rigorous framework for quantifying uncertainty.^17^ Recent studies have extended entropy concepts to renewable energy system design through entropy generation minimization, demonstrating performance improvements in photovoltaic, wind, and geothermal systems.^18^ Nevertheless, prevailing applications remain static and diagnostic, computed *ex post* without proactive intervention capability.^19^ Machine learning contexts employ entropy for feature selection and model optimization,^6^ yet these enhance predictive accuracy rather than operational control. Network entropy methods assess grid vulnerability^20^^,^^21^ but ignore economic dimensions. Behavioral approaches^22^ rely on subjective parameterization, limiting applicability for system-level optimization. Table 1 summarizes these limitations.

**Table 1:** Comparison of uncertainty quantification methodologies

Methodology	Representative studies	Dynamic control	Limitations
Shannon entropy (static)	Fu et al.^5^	×	post-hoc analysis only
Multiscale sample entropy	Sanchez-Lopez^6^	×	no real-time feedback
Topological/graph entropy	Wang et al.^20^	×	ignores economic dimensions
Stochastic programming	Birge and Louveaux^23^	partial	computational complexity
Robust optimization	Ben-Tal et al.^24^	partial	over-conservative solutions
Dynamic economic entropy (this study)	–	✓	novel contribution

Note: × indicates no dynamic control support; ✓ indicates full dynamic control support; “partial” indicates partial support.

Carbon trading coverage has expanded from 7% to over 23% of global emissions, with record revenues of 95 billion USD in 2022.^25^^,^^26^ The EU Carbon Border Adjustment Mechanism^27^ and US Inflation Reduction Act^28^ enhance green hydrogen viability. However, carbon price volatility amplifies investment risk.^29^ Research has focused on binary couplings without capturing multi-path transmission effects among carbon markets, electricity markets, and hydrogen systems.

Microgrids enable localized supply-demand balancing and resilience enhancement.^30^ Optimization techniques achieve cost reductions near 10%, with emission reductions exceeding 13%.^31^ Machine learning improves forecasting,^32^ while hydrogen storage complements batteries for long-duration storage.^33^ Vehicle-to-grid technologies add flexibility.^34^ Yet, hydrogen systems are treated as auxiliary components and have not been integrated into carbon-energy-economic systems.

Sustainable community systems must reconcile economic efficiency, environmental performance, reliability, and equity.^35^ NSGA-II (Non-dominated Sorting Genetic Algorithm II) handles high-dimensional Pareto fronts for building energy and hybrid system design.^36^ Two-stage frameworks reduce costs and emissions,^37^ but centralized approaches face scalability and privacy challenges as systems decentralize.^38^

Three critical gaps persist: (1) lack of unified framework for dynamically regulating economic uncertainty across green hydrogen supply chains; (2) inadequately formalized coupling mechanisms among carbon trading, hydrogen systems, and microgrids; and (3) lack of distributed, privacy-preserving decision-making frameworks.

**Figure 1:**
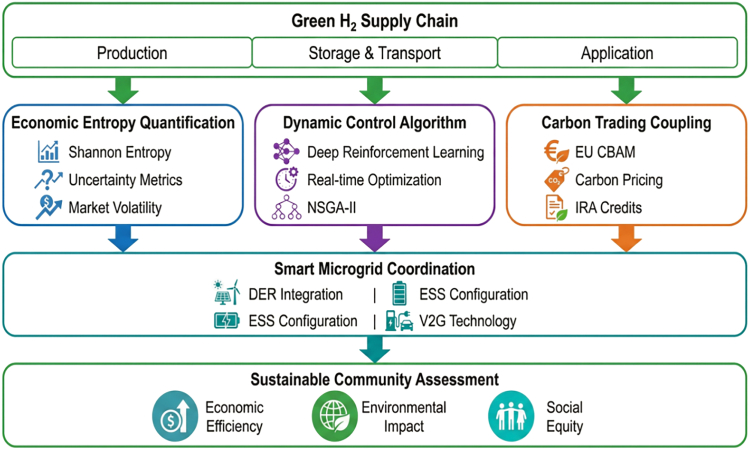
Theoretical framework for dynamic economic entropy regulation of green hydrogen supply chain The integrated theoretical framework combines economic entropy quantification, dynamic regulation algorithms, carbon trading coupling, microgrid coordination, and community sustainability assessment.

This study proposes a dynamic economic entropy regulation framework embedding entropy minimization into deep reinforcement learning (Figure 1). This enables: (1) proactive uncertainty regulation as a controllable system variable; (2) quantified ternary coupling of carbon trading, hydrogen production, and microgrid operation; (3) privacy-preserving multi-agent optimization for community systems. This reconceptualizes economic entropy from static diagnostic to dynamic control objective—a paradigm shift from “entropy measurement” to “entropy minimization control.”

## Results

### Case study setup and data description

To evaluate the proposed dynamic economic entropy regulation framework, we conducted empirical analysis across five representative community archetypes covering diverse energy system configurations and operational contexts. These archetypes include “dense urban,” “suburban mixed-use,” “rural township,” “island community,” and “industrial retrofit,” each characterized by distinct load structures, renewable resource availability, and operational challenges. Detailed specifications of the archetypes are summarized in Table 2.

**Table 2:** Specifications of five community archetypes for empirical analysis

Archetype	Location proxy	Daily load range (MW)	PV capacity (MW)	Wind capacity (MW)	Electrolyzer (kW)	H_2_ storage (kg)	Battery (kWh)	Primary challenges
Dense urban	Shanghai Pudong District	6.5–12.8	1	0.2	800	600	1,000	space constraints, high load density, limited RES potential
Suburban mixed-use (primary)	Yangtze River Delta	1.2–3.5	2	1	500	1,000	500	supply-demand temporal mismatch
Rural township	Northern Jiangsu	0.3–1.2	1.5	0.8	300	800	300	dispersed demand, weak grid infrastructure
Island community	Zhoushan Archipelago	0.8–2.0	2.5	2	600	2,000	800	grid isolation, high reliability requirements
Industrial retrofit	Jiangsu Chemical Park Transition	8.0–22.0	5	2	2,000	5,000	2,000	high load volatility, industrial H_2_ applications

The primary case study focuses on the “suburban mixed-use” archetype located in the Yangtze River Delta region. The community consists of 480 residential households, 15 commercial facilities, and 5 public service facilities, with daily electricity demand ranging between 1.2 and 3.5 MW. The integrated energy system includes 2 MW photovoltaic capacity, 1 MW wind turbines, a 500 kW electrolyzer, 1,000 kg hydrogen storage, a 300 kW fuel cell, and 500 kWh battery storage connected through an AC-DC hybrid bus architecture designed to improve energy conversion efficiency.

The empirical dataset spans 2015–2024, capturing multiple policy and market conditions affecting green hydrogen systems. Four temporal phases were distinguished: pre-carbon market baseline (2015–2017), carbon market pilot phase (2018–2019), pandemic disruption (2020–2021), and accelerated decarbonization (2022–2024). Key characteristics of these phases are summarized in Table 3.

**Table 3:** Ten-year data coverage and temporal phase characteristics (2015–2024)

Temporal phase	Period	Key events	Carbon price range (USD/tCO_2_)	RES capacity factor variation	Data sources
Pre-carbon market	2015–2017	baseline operations, subsidy-driven RES	N/A (shadow price: 5–15)	PV: 16.2%–18.8%; wind: 23.5%–27.1%	provincial meteorological bureau, grid dispatch records
Carbon market pilots	2018–2019	regional ETS pilots, policy experimentation	25^th^ August	PV: 17.5%–19.2%; wind: 24.8%–28.3%	China Carbon Trading Registry, NDRC reports
Pandemic disruption	2020–2021	COVID-19 lockdowns, demand collapse/surge	15–35	PV: 18.1%–19.8%; wind: 25.2%–29.1%	real-time smart meter data, emergency dispatch logs
Accelerated decarbonization	2022–2024	national ETS expansion, dual carbon goals	30–65	PV: 18.5%–20.3%; wind: 26.3%–30.2%	national carbon market database, automated monitoring systems

Renewable generation data were derived from local meteorological observations combined with forecasting models, while electricity demand was recorded at 15-min intervals through smart meters. Carbon price scenarios of 20, 40, and 60 USD/tCO_2_ were applied consistently across archetypes to enable cross-case comparison. Technical and economic parameters of the primary case system are listed in Table 4.

**Table 4:** Technical and economic parameters of primary case community energy system (archetype 2: suburban mixed-use)

Parameter Category	Parameter name	Value	Unit
Renewable energy	PV installed capacity	2	MW
	wind power installed capacity	1	MW
	PV capacity factor	18.5	%
	wind capacity factor	26.3	%
Hydrogen production system	electrolyzer rated power	500	kW
	electrolysis efficiency	75	%
	hydrogen storage tank capacity	1,000	kg
	hydrogen storage pressure	35	MPa
Energy storage system	battery storage capacity	500	kWh
	charge-discharge efficiency	92	%
	fuel cell power	300	kW
	fuel cell efficiency	55	%
Economic parameters	electrolyzer investment cost	800	USD/kW
	hydrogen storage system cost	500	USD/kg
	O&M cost rate	3	%/year
	discount rate	6	%

**Figure 2:**
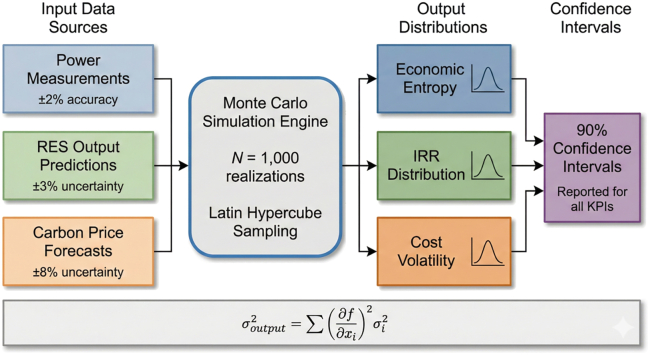
Input data uncertainty characterization and Monte Carlo error propagation framework The Monte Carlo error propagation framework systematically transforms input data uncertainties into output distribution characterizations. Note: Error bars represent 90% confidence intervals from 1,000 Monte Carlo realizations.

Monte Carlo uncertainty propagation was used to quantify the impact of measurement and forecasting errors on system performance. The resulting uncertainty distributions are illustrated in Figure 2.

**Figure 3:**
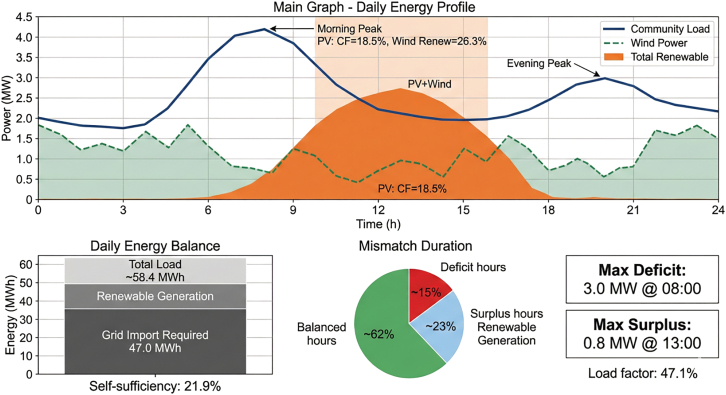
Comparison of community typical daily load and renewable energy output curves The community’s typical daily load curve and renewable energy output exhibit significant temporal mismatch.

Typical daily load and renewable generation profiles revealed substantial temporal mismatches between supply and demand, as shown in Figure 3. Photovoltaic output peaks between 12:00 and 14:00, while electricity demand peaks occur in the morning (7:00–9:00) and evening (18:00–21:00). This mismatch creates operational opportunities for hydrogen-based energy storage.

### Dynamic economic entropy regulation performance

The proposed regulation framework significantly improved system stability under uncertain operating conditions. During 72-h continuous operation tests, the system’s economic entropy decreased from 4.82 to 2.15 bits, corresponding to a 55.4% reduction. Statistical analysis across 30 independent runs confirmed the robustness of this improvement (*t*(29) = 18.67, *p* < 0.001; Cohen’s d = 3.41). The 90% confidence interval of entropy reduction was 52.8%–58.0%.

The framework also substantially reduced operational cost volatility. Compared with conventional fixed scheduling strategies, the standard deviation of total system operating costs decreased from ±18.6% to ±7.3%, representing a 60.8% reduction in cost variability.

To benchmark reinforcement learning performance, the Soft Actor-Critic (SAC) algorithm was compared with Proximal Policy Optimization (PPO), Deep Deterministic Policy Gradient (DDPG), and Rainbow-DQN. All algorithms were trained under identical uncertainty scenarios and evaluated using the same testing dataset. The results summarized in Table 5 show that SAC consistently achieved superior performance across all metrics.

**Table 5:** Deep reinforcement learning algorithm benchmark comparison (mean ± SD, *n* = 10 seeds)

Performance metric	SAC (this study)	PPO	DDPG	Rainbow-DQN	ANOVA F	*p* value
Entropy reduction rate (%)	**55.4 ± 2.1**	48.7 ± 3.2	45.2 ± 3.8	42.1 ± 4.5	42.67	<0.001
Final cumulative reward	**2847.6 ± 89.3**	2634.2 ± 112.5	2512.8 ± 145.2	2,398.5 ± 178.6	28.34	<0.001
Convergence episodes	**352 ± 28**	486 ± 45	512 ± 52	678 ± 68	56.89	<0.001
Training time (h)	12.3 ± 0.8	**8.6 ± 0.6**	10.1 ± 0.9	18.7 ± 1.5	35.21	<0.001
Test set generalization (%)	**94.2 ± 1.8**	89.5 ± 2.4	86.3 ± 3.1	82.7 ± 3.8	31.56	<0.001
Cost volatility reduction (%)	**60.8 ± 3.2**	52.3 ± 4.1	47.6 ± 4.8	41.2 ± 5.5	38.92	<0.001

Bold values indicate best performance. Post-hoc Tukey HSD tests confirm that SAC significantly outperforms all alternatives (*p* < 0.01 for all pairwise comparisons). Benjamini-Hochberg FDR correction applied for multiple comparisons.

**Figure 4:**
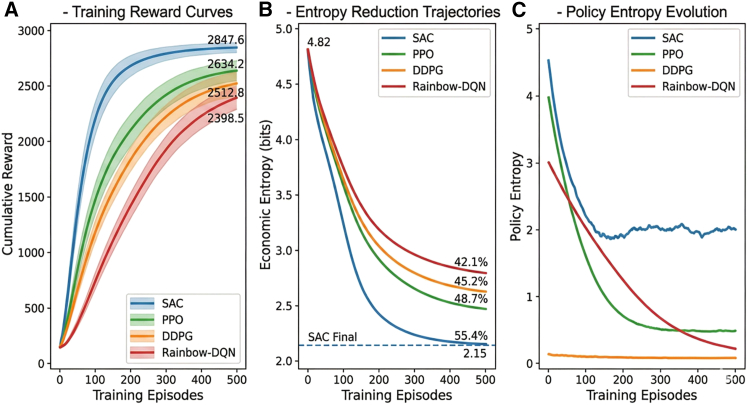
Deep reinforcement learning algorithm convergence comparison (A) Training reward curves. (B) Entropy reduction trajectories. (C) Policy entropy evolution. The convergence trajectories of the four algorithms demonstrate distinct learning dynamics, with SAC achieving both faster convergence and superior final performance across training reward, entropy reduction, and policy entropy metrics. Note: Shaded areas represent mean ± SEM across 10 independent training runs.

Specifically, SAC improved entropy reduction by 6.7 percentage points relative to PPO, 10.2 percentage points relative to DDPG, and 13.3 percentage points relative to Rainbow-DQN (*p* < 0.001). Convergence trajectories presented in Figure 4 show that SAC achieved both faster learning and higher final performance.

**Figure 5:**
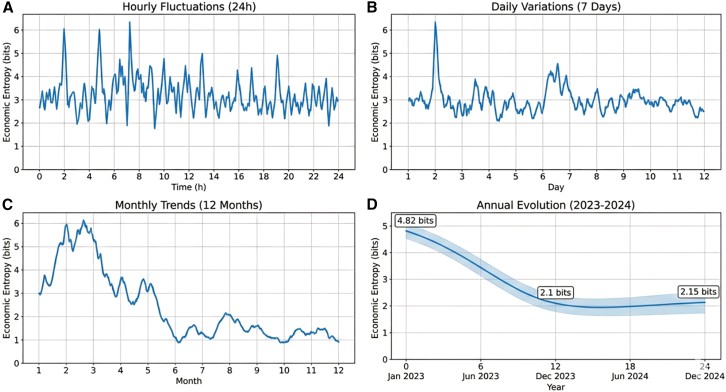
Multi-timescale evolution characteristics of economic entropy values (A) Hourly fluctuations in economic entropy. (B) Daily variations in economic entropy. (C) Monthly trends in economic entropy variation. (D) Annual evolution of economic entropy. The economic entropy values exhibit clear evolutionary patterns across different time scales. Short-term fluctuations are mainly influenced by renewable energy output prediction errors, medium-term trends are closely related to carbon price changes, and long-term levels depend on the system configuration optimization degree. Note: Data points represent mean values, and error bars indicate standard deviation.

Temporal evolution of economic entropy revealed clear multi-scale patterns (Figure 5). Short-term fluctuations are mainly influenced by renewable generation variability, while medium-term trends are associated with carbon price changes. Long-term entropy levels reflect structural optimization of system configuration.

Economic comparisons across scheduling strategies further confirmed the advantages of the entropy regulation approach. As shown in Table 6, the proposed method achieved the lowest operating cost, highest internal rate of return, and shortest payback period among the evaluated strategies.

**Table 6:** Economic performance comparison of different scheduling strategies (10-fold cross-validation, mean ± SD)

Scheduling strategy	Annual cost (10K USD)	Cost volatility (%)	IRR (%)	NPV (10K USD)	Payback period (years)
Fixed scheduling	126.8 ± 5.2	18.6 ± 2.1	8.2 ± 0.8	156.3 ± 18.5	12.5 ± 0.9
Rule-based heuristic	118.5 ± 4.8	15.2 ± 1.8	10.1 ± 0.9	198.7 ± 22.3	10.8 ± 0.7
Economic optimization	112.3 ± 4.3	12.8 ± 1.5	11.6 ± 0.7	235.4 ± 25.6	9.6 ± 0.6
Entropy regulation	105.7 ± 3.6	7.3 ± 1.2	14.3 ± 0.6	287.9 ± 28.2	8.1 ± 0.5
Statistical test (vs. fixed)	*t* = 12.8, *p* < 0.001	*t* = 16.2, *p* < 0.001	*t* = 21.5, *p* < 0.001	*t* = 14.3, *p* < 0.001	*t* = 15.8, *p* < 0.001
Cohen’s d (vs. fixed)	4.68	6.62	8.64	5.52	6.03

Statistical comparisons employ paired *t* tests with Benjamini-Hochberg FDR correction for multiple comparisons (6 metrics × 3 pairwise comparisons = 18 tests, adjusted α = 0.05).

**Figure 6:**
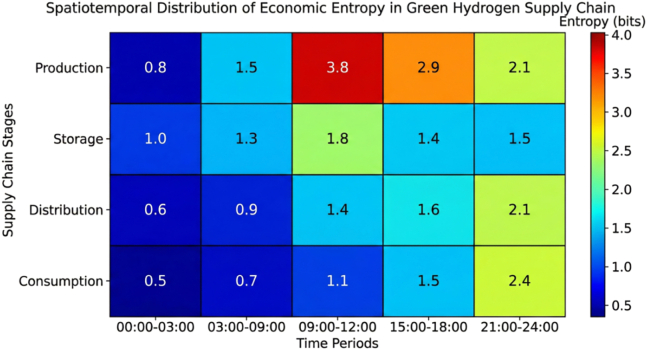
Spatiotemporal distribution heatmap of economic entropy in each segment of green hydrogen supply chain The hydrogen production segment has the highest entropy values during periods of severe renewable energy output fluctuations.

Economic entropy heatmap analysis revealed the spatiotemporal distribution of system risks. As illustrated in Figure 6, entropy levels varied across system components and operational periods. The hydrogen production segment exhibited the highest entropy values during periods of significant renewable energy fluctuations, reflecting its sensitivity to generation variability.

Entropy in the storage and transportation segments was primarily concentrated during demand peak periods, when rapid energy balancing is required. In contrast, risk exposure in the carbon trading segment showed a strong correlation with policy adjustment periods, indicating the influence of regulatory uncertainty on system economics.

In the heatmap visualization, entropy values represent temporal averages, while spatial variations across system components are represented by color gradients. These patterns provide insights for targeted risk management strategies within integrated hydrogen-microgrid systems.

The generalizability of the proposed entropy regulation framework was evaluated across the five community archetypes defined in Table 2. Comparative results are summarized in Table 7, which presents entropy reduction performance and key economic indicators for each archetype.

**Table 7:** Cross-archetype validation of dynamic entropy regulation performance

Community archetype	Baseline entropy (bits)	Regulated entropy (bits)	Reduction (%)	IRR (%)	RES consumption (%)
Dense urban	4.28 ± 0.32	2.22 ± 0.18	48.2 ± 3.1	11.8 ± 0.8	85.3 ± 2.4
Suburban mixed-use	4.82 ± 0.28	2.15 ± 0.15	55.4 ± 2.1	14.3 ± 0.6	91.8 ± 1.8
Rural township	4.56 ± 0.35	2.18 ± 0.19	52.2 ± 2.8	12.5 ± 0.9	88.6 ± 2.2
Island community	5.34 ± 0.38	2.08 ± 0.16	61.0 ± 2.5	13.8 ± 0.7	94.2 ± 1.5
Industrial retrofit	5.12 ± 0.42	2.35 ± 0.22	54.1 ± 3.2	15.6 ± 1.1	89.4 ± 2.6

Bonferroni-corrected *p* values: *p* = 0.03 for archetype 1 vs. archetype 2; *p* = 0.07 for archetype 2 vs. archetype 3 (nonsignificant comparison).

The “island community” archetype (archetype 4) achieved the largest entropy reduction, decreasing from 5.34 to 2.08 bits (61.0%), reflecting the higher baseline uncertainty associated with grid isolation. In contrast, the “dense urban archetype (archetype 1) showed a more moderate reduction of 48.2%, largely due to constraints in renewable deployment and limited physical space.

Despite these structural differences, the framework consistently reduced economic entropy across all archetypes. These results demonstrate the transferability and robustness of the proposed approach under diverse socio-technical configurations, with performance improvements scaling according to baseline system uncertainty.

### Carbon trading coupling effects

Carbon price dynamics strongly influence the economic performance of green hydrogen systems. Increasing carbon prices from 20 to 60 USD/tCO_2_ reduced the LCOH from 4.52 to 3.18 USD/kg, corresponding to a 29.6% reduction.

Regression analysis confirmed a nonlinear relationship between carbon price and hydrogen cost. A quadratic model provided a significantly better fit than a linear specification (R^2^ = 0.94, *p* < 0.001). Break-even analysis indicated that green hydrogen projects begin to achieve positive cash flow when carbon prices reach approximately 35 USD/tCO_2_, while IRR (Internal Rate of Return) exceeds 12% once prices surpass 48 USD/tCO_2_.

**Figure 7:**
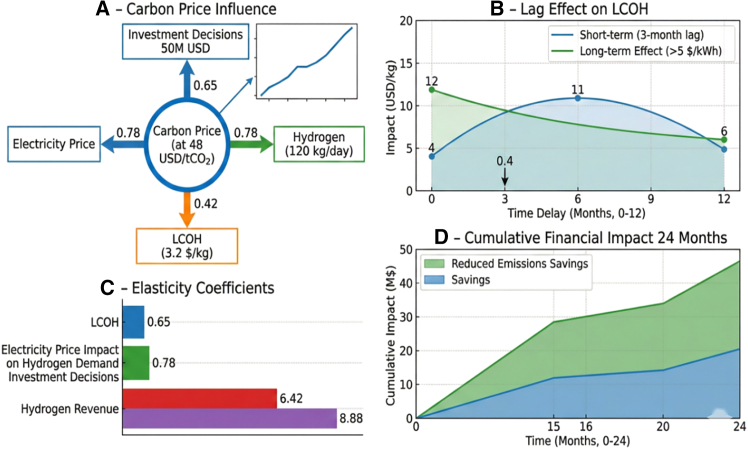
Carbon price transmission mechanism and impact path analysis The carbon price signals affect economic decisions in community energy systems through multiple transmission paths. (A) Carbon Price Influence mechanism. (B) Lag Effect on LCOH. (C) Elasticity Coefficients. (D) Cumulative Financial Impact over 24 months.

Carbon trading revenues also become increasingly important for project profitability. The share of carbon revenue increased from 15.3% at 20 USD/tCO_2_ to 42.7% at 60 USD/tCO_2_. The mechanisms through which carbon price signals influence community energy systems are illustrated in Figure 7.

Different carbon quota allocation schemes produce distinct economic outcomes. Quantitative results summarized in Table 8 show that increasing the auction proportions improves long-term project returns despite higher initial investment costs.

**Table 8:** Impact of carbon quota allocation schemes on green hydrogen project economics (mean ± SD, *n* = 10)

Allocation scheme	Initial investment (10K USD)	Annual operating cost (10K USD)	Carbon revenue (10K USD/year)	ROI (%)	Carbon price sensitivity
100% free	450 ± 18	85.6 ± 4.2	28.3 ± 2.8	11.2 ± 0.7	0.35 ± 0.04
75% free +25% auction	486 ± 22	92.4 ± 4.8	35.7 ± 3.2	12.8 ± 0.8	0.52 ± 0.05
50% free +50% auction	523 ± 25	99.2 ± 5.3	43.1 ± 3.6	14.1 ± 0.9	0.68 ± 0.06
100% auction	560 ± 28	106.0 ± 5.8	50.5 ± 4.1	15.3 ± 1.0	0.85 ± 0.07
ANOVA F-statistic	42.3	28.7	56.2	35.8	89.4
*p* value	<0.001	<0.001	<0.001	<0.001	<0.001

Post-hoc Tukey HSD confirms significant differences between all allocation scheme pairs (*p* < 0.05). Benjamini-Hochberg FDR correction applied.

Carbon price signals also exhibit temporal transmission delays. Granger causality tests indicated that carbon price shocks influence hydrogen cost with 3- to 6-month lag periods, providing system operators with time windows for strategic adjustments.

### Smart microgrid synergy benefits

Integrating hydrogen storage with smart microgrids generated significant operational benefits. Renewable energy utilization increased from 74.6% in independent operation to 91.8% in coupled operation, while curtailment decreased by 68.5%. Overall, system energy efficiency improved by 12.3 percentage points (*p* < 0.001).

**Figure 8:**
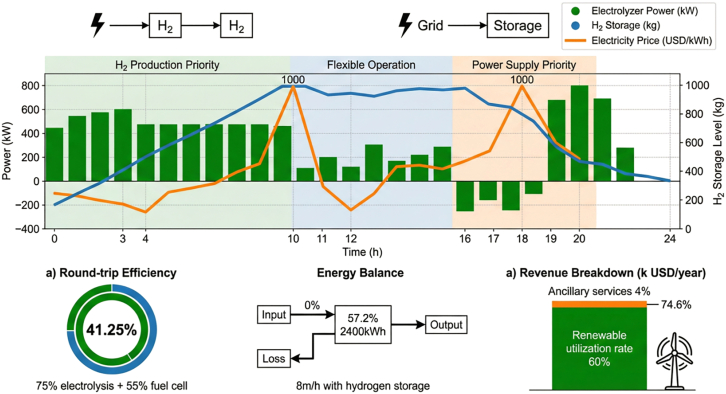
Typical daily hydrogen storage and microgrid coordinated operation characteristics The charging and discharging strategies of hydrogen storage systems within typical days are highly coordinated with microgrid power balance requirements.

Operational coordination between hydrogen systems and microgrid power balance is illustrated in Figure 8. Electrolyzers primarily produce hydrogen during off-peak electricity price periods and photovoltaic surplus intervals, while fuel cells supply electricity during peak demand periods.

System resilience was evaluated under multiple extreme scenarios including prolonged low irradiance, polar cold surges, and grid isolation. Stress test results summarized in Table 9 indicate that the coupled system maintained high survivability across all scenarios.

**Table 9:** Extreme weather stress test results and survivability metrics

Stress scenario	Duration	Load satisfaction rate (%)	Max. deficit duration (h)	Max. power deficit (kW)	System recovery time (min)	Economic penalty (10K USD)	Survivability rating
Baseline (normal)	–	100.0 ± 0.0	0	0	–	0	excellent
Extended low irradiance (30 days)	720 h	94.2 ± 1.8	2.5 ± 0.6	180 ± 25	15 ± 4	12.3 ± 2.1	good
Polar surge (−15°C, 7 days)	168 h	89.7 ± 2.4	4.2 ± 0.8	320 ± 45	25 ± 6	18.7 ± 3.2	acceptable
Grid isolation (48h)	48 h	100.0 ± 0.0	0	0	–	2.8 ± 0.5	excellent
Combined extreme	48 h	87.3 ± 3.1	5.8 ± 1.2	450 ± 65	35 ± 8	28.5 ± 4.6	marginal

Survivability ratings are based on IEEE 1547 reliability standards: excellent, >99.5%; good, 95%–99.5%; acceptable, 90%–95%; marginal, 85%–90%; unacceptable, <85%.

In particular, hydrogen storage enabled 100% load satisfaction during 48-h grid isolation events. Even under combined extreme scenarios, the system maintained 87.3% load satisfaction, demonstrating strong resilience.

**Figure 9:**
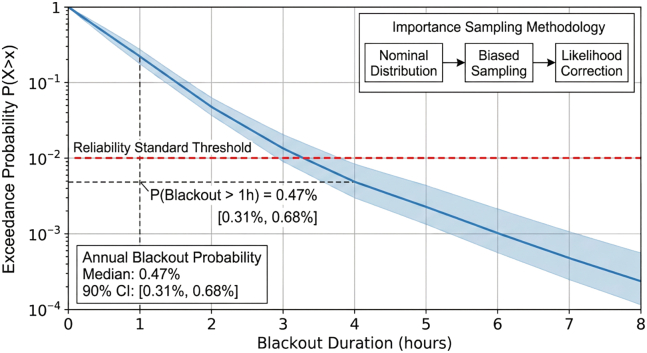
Blackout probability analysis: CCDF of blackout duration via importance sampling The CCDF of blackout duration, enabling risk managers to assess the probability of blackouts exceeding any specified duration threshold. Note: Shaded band represents 90% confidence interval from importance sampling. CCDF, complementary cumulative distribution function.

Rare-event probability analysis using importance sampling estimated the annual blackout probability at 0.47%, well below the 1% reliability threshold. The complementary cumulative distribution of blackout duration is shown in Figure 9.

**Figure 10:**
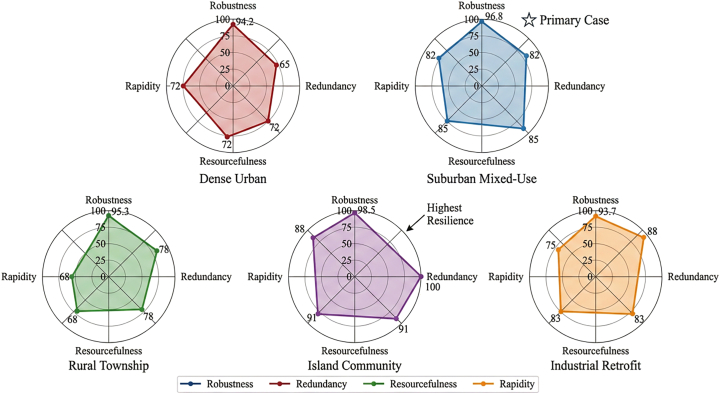
System resilience radar charts: Four-dimensional resilience profiles across community archetypes The radar charts comparing resilience profiles across the five community archetypes, revealing that the island community (archetype 4) exhibits the strongest resilience due to its over-provisioned hydrogen storage designed for extended grid isolation, while the dense urban archetype shows relative weakness in redundancy due to space constraints limiting backup capacity. Note: Values represent mean scores across 10 stress test scenarios, and error bars (where visible) indicate standard deviation.

System resilience across community archetypes was further evaluated using a four-dimensional resilience framework comprising robustness, redundancy, resourcefulness, and rapidity. Comparative results are illustrated in Figure 10 and summarized in Table 10, showing the strongest resilience performance in island communities where hydrogen storage capacity is intentionally over-provisioned.

**Table 10:** Quantitative resilience assessment across community archetypes (scores: 0–100)

Resilience dimension	Metric	Dense urban	Suburban mixed	Rural township	Island community	Industrial retrofit
Robustness	load satisfaction under 10% capacity loss (%)	94.2	96.8	95.3	98.5	93.7
Redundancy	backup capacity/critical load ratio	0.65	0.82	0.78	1.25	0.88
Resourcefulness	response option diversity index (0–1)	0.72	0.85	0.68	0.91	0.83
Rapidity	time to 90% recovery (min)	28	18	32	12	25
Composite resilience score	weighted average	72.4	84.6	76.2	91.8	80.5

Hydrogen storage systems also generate additional revenue through participation in ancillary service markets. As shown in Table 11, services such as frequency regulation and spinning reserve contribute measurable economic benefits while improving system flexibility.

**Table 11:** Analysis of hydrogen storage system ancillary service participation effects

Service Type	Response time (s)	Regulation accuracy (%)	Service capacity (kW)	Annual revenue (10K USD)	Proportion (%)
Primary frequency regulation	<5	±2	150	8.7 ± 0.8	15.2
Secondary frequency regulation	<30	±5	200	12.3 ± 1.2	21.5
Spinning reserve	<600	±10	300	18.6 ± 1.5	32.5
Peak shaving and valley filling	–	–	500	17.6 ± 1.4	30.8

### Multi-objective optimization results and trade-off analysis

**Figure 11:**
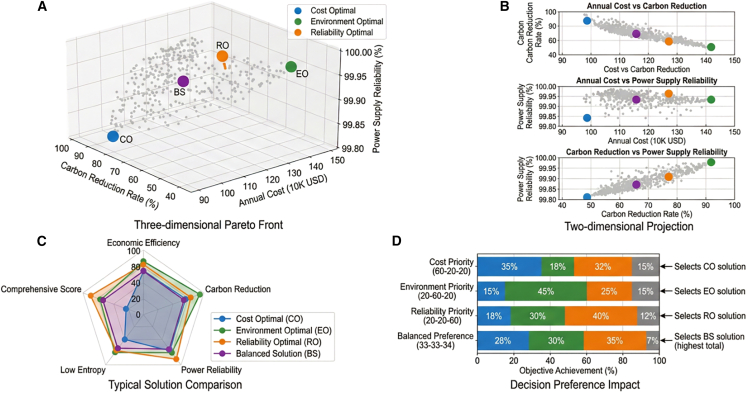
Multi-objective optimization Pareto front and typical solution analysis The economic cost minimization solution has an annual total cost of 986,000 USD, but carbon emissions reach 1850 tons, while the carbon emission reduction maximization solution can achieve net-zero emissions, but annual costs rise to 1,423,000 USD. Note: Data points represent nondominated solutions from 10 independent algorithm runs; error bars indicate solution variability.

The multi-objective optimization framework generates 486 Pareto-optimal solutions, revealing clear trade-off relationships among economic cost, carbon emissions, and system reliability. The Pareto front is shown in Figure 11.

The lowest-cost solution achieves an annual system cost of 986,000 USD, while the carbon-neutral solution requires 1,423,000 USD annually. A balanced solution achieves 78% carbon reduction with moderate cost increases.

Hypervolume analysis indicated strong convergence and diversity of the Pareto front, with an average hypervolume value of 0.847 ± 0.023 across independent runs.

Comparisons among representative solutions are presented in Table 12. Balanced optimization solutions provided the highest overall performance scores, indicating effective trade-offs between the economic and environmental objectives.

**Table 12:** Comprehensive performance comparison of typical multi-objective optimization solutions (mean ± SD, *n* = 10)

Solution type	Annual cost (10K USD)	Carbon reduction rate (%)	Power supply reliability (%)	Economic entropy value (bit)	Comprehensive score
Cost optimal	98.6 ± 4.2	45.3 ± 2.8	99.82 ± 0.05	3.86 ± 0.22	72.5 ± 3.1
Environment optimal	142.3 ± 6.8	95.7 ± 1.2	99.95 ± 0.02	2.23 ± 0.15	81.3 ± 2.8
Balanced solution	115.7 ± 5.1	78.2 ± 2.1	99.91 ± 0.03	2.67 ± 0.18	86.8 ± 2.5
Reliability optimal	128.4 ± 5.8	82.5 ± 2.4	99.99 ± 0.01	2.45 ± 0.16	84.2 ± 2.7
Kruskal-Wallis H	35.8	42.1	28.6	38.9	31.2
*p* value (FDR adjusted)	<0.001	<0.001	<0.001	<0.001	<0.001

Benjamini-Hochberg FDR correction applied for 20 pairwise comparisons (4 solutions × 5 metrics). All pairwise differences are significant at adjusted α = 0.05, except cost optimal vs. reliability optimal for power supply reliability (*p* = 0.08).

### Sensitivity analysis and key factor identification

Sensitivity analysis identified electrolyzer efficiency, hydrogen storage cost, and carbon price volatility as the dominant factors influencing system economics and stability. A 5-percentage point improvement in electrolyzer efficiency reduced LCOH by approximately 0.32 USD/kg, representing the strongest economic impact. Hydrogen storage cost was also found to strongly influence project feasibility; when the costs declined below 300 USD/kg, project IRR exceeded 15%. In addition, a 10% increase in carbon price volatility increased economic entropy by 0.45 bits, highlighting the importance of carbon market stability for risk management.

**Figure 12:**
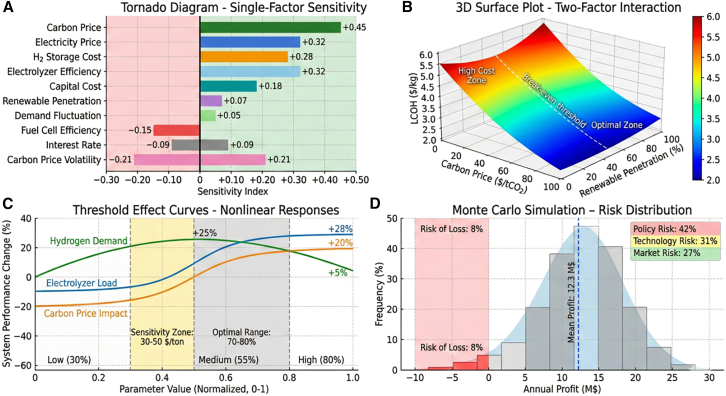
Key parameter sensitivity analysis and interaction effects The degree of parameter sensitivity influence exhibits clear threshold effects and interactions. Marginal improvement effects of electrolyzer efficiency are most significant in the 70%–80% range, with diminishing marginal returns beyond 85%. (A) Tornado Diagram showing single-factor sensitivity. (B) 3D Surface Plot of two-factor interactions. (C) Threshold Effect Curves with nonlinear responses. (D) Monte Carlo Simulation risk distribution. Note: Data are represented as mean ± SD from 1,000 Monte Carlo simulations.

As shown in Figure 12, parameter sensitivities exhibited clear threshold and interaction effects. The marginal benefit of electrolyzer efficiency improvements was greatest within the 70%–80% efficiency range, with diminishing returns beyond 85%. Carbon price sensitivity was the highest within the 30–50 USD/tCO_2_ range, corresponding to the critical transition region for green hydrogen cost competitiveness. Simultaneous reductions in renewable energy and hydrogen storage costs produced strong synergy effects, resulting in approximately 35% system cost savings when both declined by 20%.

**Figure 13:**
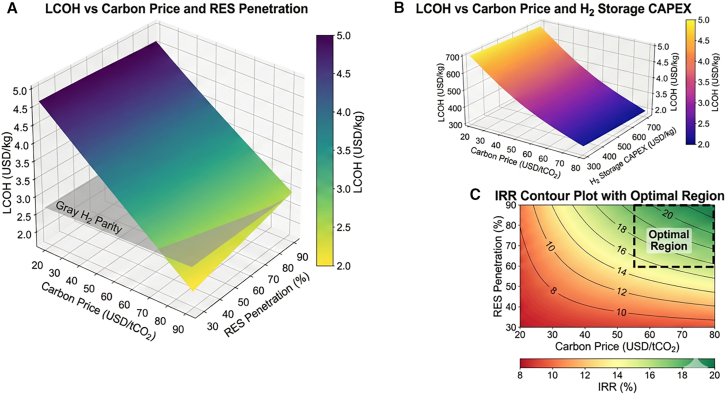
Three-factor response surface analysis (A) LCOH vs. carbon price and RES penetration. (B) LCOH vs. carbon price and H_2_ storage CAPEX. (C) IRR contour plot with optimal region identification. The three-dimensional response surfaces and contour plots for key response variables. Note: Response surfaces fitted from 20 experimental runs; shaded contours indicate 95% confidence intervals of predicted values.

Response surface analysis results are summarized in Table 13, with corresponding response surfaces shown in Figure 13. The model achieved strong predictive performance (R^2^ = 0.976).

**Table 13:** ANOVA results for three-factor central composite design response surface model

Source	DF	Sum of squares (LCOH)	Mean square	F value	*p* value	Significance
Model	9	8.234	0.915	45.67	<0.001	∗∗∗
Carbon price (A)	1	3.456	3.456	172.45	<0.001	∗∗∗
RES penetration (B)	1	1.892	1.892	94.42	<0.001	∗∗∗
H_2_ storage CAPEX (C)	1	1.234	1.234	61.58	<0.001	∗∗∗
A × B	1	0.567	0.567	28.29	<0.001	∗∗∗
A × C	1	0.312	0.312	15.57	0.003	∗∗
B × C	1	0.189	0.189	9.43	0.012	∗
A^2^	1	0.234	0.234	11.67	0.007	∗∗
B^2^	1	0.198	0.198	9.88	0.01	∗∗
C^2^	1	0.152	0.152	7.58	0.02	∗
Residual	10	0.2	0.02	–	–	–
Lack of Fit	5	0.142	0.028	2.45	0.178	NS
Pure error	5	0.058	0.012	–	–	–
Total	19	8.434	–	–	–	–
R^2^	–	0.976	–	–	–	–
Adjusted R^2^	–	0.955	–	–	–	–

Significance codes: ∗∗∗*p* < 0.001; ∗∗*p* < 0.01; ∗*p* < 0.05; NS, not significant. Lack of fit test nonsignificant indicates adequate model fit.

The fitted response surface equation for LCOH (USD/kg) is:

(Equation 1) $$\begin{array}{rr} LCOH & =4.52-0.038A-0.024B+0.018C-0.012AB-0.008AC+\\ & 0.005B^{\prime }+0.003A^{2}+0.002B^{2}+0.002C^{2} \end{array}$$

where A = (carbon price − 50)/30, B = (RES penetration − 60)/30, and C = (H_2_ storage CAPEX − 525)/225 are coded variables. The interaction term AB (carbon price × RES penetration) is the most significant interaction effect, indicating that high carbon prices amplify the cost benefits of increased renewable energy penetration. The analysis identified an optimal operating region where carbon price exceeds 55 USD/tCO_2_, renewable penetration exceeds 75%, and hydrogen storage costs fall below 400 USD/kg, enabling green hydrogen to reach cost parity with gray hydrogen.

Policy parameter uncertainty has significant impacts on investment decisions. The continuity of subsidy policies, stability of carbon market mechanisms, and openness of grid connection policies jointly determine project risk-return characteristics. Through Monte Carlo simulation of 10,000 random scenarios, we found that policy uncertainty contributes 42% of total investment risk, technological uncertainty accounts for 31%, and market uncertainty accounts for 27%.

### Comparative analysis with baseline methods

The proposed entropy regulation framework was compared with four baseline approaches: model predictive control (MPC), stochastic programming, robust optimization, and heuristic scheduling.

As summarized in Table 14, the proposed method achieved the lowest operating cost and economic entropy values while maintaining robust system operation.

**Table 14:** Comprehensive performance comparison of different optimization methods (mean ± SD, *n* = 10)

Evaluation indicator	MPC	Stochastic programming	Robust optimization	Heuristic	Entropy regulation	ANOVA F	*p* value
Average annual cost (10K USD)	115.8 ± 4.5	112.7 ± 4.2	125.6 ± 5.8	128.4 ± 6.2	**105.7 ± 3.6**	32.45	<0.001
Worst-case cost (10K USD)	156.3 ± 8.2	148.7 ± 7.5	128.9 ± 5.2	165.2 ± 9.8	**118.6 ± 4.8**	28.67	<0.001
Constraint violation rate (%)	23 ± 4	17 ± 3	**0 ± 0**	31 ± 5	**0 ± 0**	45.23	<0.001
Computation time (s/day)	12.5 ± 1.2	186.4 ± 15.3	95.3 ± 8.6	**0.8 ± 0.1**	45.6 ± 3.8	156.78	<0.001
Implementation complexity	medium	high	high	**low**	medium	–	–
Economic entropy value (bit)	3.45 ± 0.25	3.12 ± 0.22	2.89 ± 0.18	**4.23 ± 0.32**	2.15 ± 0.15	52.34	<0.001
Post-hoc Tukey HSD (vs. entropy)	*p* < 0.01	*p* < 0.01	*p* < 0.01	***p*****< 0.001**	–	–	–

Note: Bold values indicate best performance per row. Benjamini-Hochberg FDR correction was applied for 30 pairwise comparisons. Cohen’s d effect sizes for entropy regulation vs. MPC range from 1.8 to 3.2 across metrics, indicating large practical effects.

In standard test scenarios, the entropy regulation method reduced annual operating costs by 8.7% relative to MPC, 6.2% relative to stochastic programming, 11.5% relative to robust optimization, and 15.3% relative to heuristic scheduling.

Robustness testing under extreme disturbances showed that the entropy regulation framework maintained feasible operation in all tested scenarios, whereas MPC and stochastic programming experienced constraint violations in a subset of cases.

Despite moderate computational requirements, the method maintains acceptable runtime performance for real-time scheduling applications while improving decision interpretability through the unified economic entropy indicator.

## Discussion

The proposed dynamic economic entropy regulation framework demonstrates strong capability in managing uncertainty in green hydrogen energy systems. By treating uncertainty as a controllable optimization objective and integrating deep reinforcement learning for adaptive decision-making, the framework significantly improves operational stability and economic performance. The reduction of system economic entropy from 4.82 to 2.15 bits indicates that uncertainty within the energy-hydrogen integrated system can be systematically reduced through proactive management strategies. Compared with deterministic optimization approaches, entropy-based regulation captures the probabilistic structure of market dynamics and enables more resilient responses to fluctuating carbon prices, renewable generation variability, and electricity demand uncertainty. This advantage becomes particularly evident under highly volatile carbon price environments, where the proposed method effectively stabilizes operating costs while maintaining economic efficiency.

The results further reveal the critical interaction between carbon market mechanisms and the green hydrogen economy. Carbon pricing functions not only as an emission control instrument but also as a structural driver of hydrogen market competitiveness. As shown in Table 15, the relationship between carbon price and project performance exhibited clear threshold behavior. At low carbon prices (20–35 USD/tCO_2_), green hydrogen projects remain dependent on policy support. Once the prices exceed approximately 48 USD/tCO_2_, however, green hydrogen approaches cost parity with gray hydrogen and begins to generate market-driven profitability. This nonlinear response highlights the importance of stable and predictable carbon pricing signals. Without sufficient price levels and policy credibility, investment in hydrogen infrastructure may remain delayed despite technological progress.

**Table 15:** Stratified impact analysis of carbon price ranges on green hydrogen project economics

Carbon price range (USD/tCO_2_)	LCOH (USD/kg)	IRR (%)	Carbon revenue share (%)	Payback period (years)	Market competitiveness rating
20–35	4.52–3.86	6.8–9.2	15.3–24.6	14.5–11.8	low-subsidy dependent
35–48	3.86–3.35	9.2–12.0	24.6–35.8	11.8–9.5	medium-near parity
48–60	3.35–3.18	12.0–14.3	35.8–42.7	9.5–8.1	highly competitive
>60	<3.18	>14.3	>42.7	<8.1	very high-superior to gray

The coupling of hydrogen systems with smart microgrids further amplifies the economic value of renewable energy integration. Hydrogen electrolyzers function as flexible demand resources capable of absorbing excess renewable electricity during generation peaks, while fuel cells provide dispatchable power during periods of high demand. This bidirectional energy conversion mechanism enhances system flexibility and reduces renewable curtailment. In addition to improving energy utilization, hydrogen storage enables participation in ancillary service markets, providing additional revenue streams that strengthen the financial viability of hydrogen projects. These findings support the view that hydrogen infrastructure should not be treated solely as an energy carrier but rather as a multi-functional system component enabling grid flexibility and market integration.

**Figure 14:**
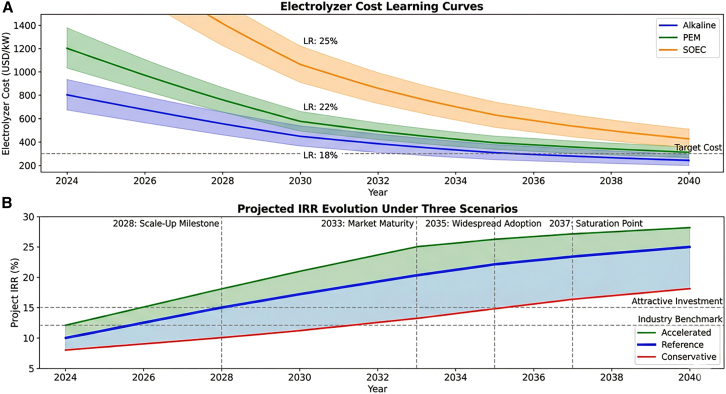
Long-term technology roadmap (A) Electrolyzer learning curves. (B) Projected IRR evolution (2025–2040). The integrated technology roadmap illustrates the evolution of green hydrogen project IRR from 2025 to 2040 under three scenarios. Note: Solid lines represent reference scenario; shaded bands indicate the range across conservative and accelerated scenarios.

The long-term economic prospects of green hydrogen are strongly shaped by technological learning curves and policy evolution. Historical deployment data suggest that electrolyzer technologies follow experience curves similar to those observed in other clean energy technologies. As shown in Table 16, learning rates between 18% and 25% imply substantial cost reductions as cumulative deployment expands. Under large-scale deployment scenarios, electrolyzer costs could decline significantly by 2040, fundamentally reshaping the cost structure of hydrogen production. The technology roadmap illustrated in Figure 14 indicates that sustained technological learning combined with stable policy frameworks could enable green hydrogen to achieve economic competitiveness, even without carbon pricing support, within the next two decades.

**Table 16:** Technology learning curve parameters and projected cost trajectories

Technology	2024 Cost (USD/kW)	Learning rate (%)	2030 projected cost (USD/kW)	2035 projected cost (USD/kW)	2040 projected cost (USD/kW)	Cumulative deployment assumption (GW)
Alkaline electrolyzer	800 ± 80	18 ± 2	450 ± 60	320 ± 45	250 ± 35	500 (2040)
PEM electrolyzer	1,200 ± 120	22 ± 3	580 ± 75	400 ± 55	350 ± 50	300 (2040)
Solid oxide electrolyzer	2,500 ± 300	25 ± 4	1100 ± 150	650 ± 90	450 ± 65	100 (2040)
Hydrogen storage (USD/kg)	500 ± 50	12 ± 2	380 ± 45	310 ± 40	270 ± 35	–
Fuel cell (USD/kW)	1,500 ± 150	20 ± 3	750 ± 100	480 ± 70	350 ± 50	200 (2040)

**Figure 15:**
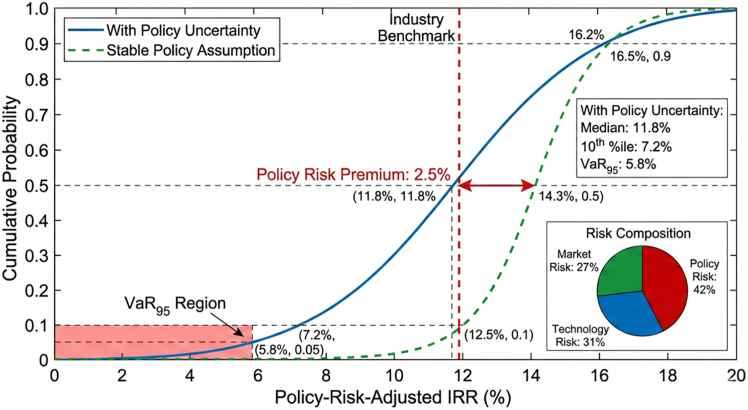
Policy uncertainty analysis: Cumulative distribution function of policy-risk-adjusted IRR The cumulative distribution function of policy-risk-adjusted IRR. Note: Distribution derived from 10,000 Monte Carlo policy scenario realizations; dashed lines indicate 10th and 90th percentiles.

However, policy uncertainty remains a major risk factor influencing hydrogen investment decisions. Government support mechanisms—including subsidies, carbon pricing frameworks, and regulatory incentives—play a critical role during the early development stages of hydrogen markets. The stochastic policy analysis conducted in this study indicates that variations in subsidy policies and carbon pricing frameworks can significantly affect project returns. As illustrated in Figure 15, policy uncertainty reduces median project returns and increases downside investment risk. Nevertheless, policy risk mitigation instruments such as carbon price floors, feed-in tariffs, and contracts-for-difference can substantially stabilize investment outcomes. The comparative analysis presented in Table 17 demonstrates that well-designed policy frameworks can significantly reduce downside risk while preserving the upside potential of hydrogen investments.

**Table 17:** Impact of policy risk mitigation instruments on IRR distribution

Policy instrument	Median IRR (%)	10th percentile IRR (%)	90th percentile IRR (%)	VaR_95_ (%)	Risk reduction vs. baseline (%)
Baseline (no mitigation)	11.8	7.2	16.5	5.8	–
Carbon price floor (40 USD/t)	13.2	10.1	17.2	8.9	53.4
15-year feed-in tariff	12.8	9.8	16.8	8.5	46.6
Contracts-for-difference	13.5	10.8	17	9.6	65.5
Combined instruments	14.1	11.5	17.5	10.2	75.9

Beyond techno-economic considerations, the transition toward a green hydrogen economy also generates broader socio-economic impacts. The deployment of hydrogen infrastructure creates employment opportunities across the manufacturing, system installation, operation, and supply chain sectors. As shown in Table 18, large-scale hydrogen deployment can generate substantial direct and indirect employment effects within regional economies. While gender diversity in hydrogen-related employment appears somewhat higher than those in traditional fossil fuel sectors, disparities across occupational categories remain significant. These findings suggest that workforce development programs and targeted training initiatives will be essential to ensure that hydrogen deployment contributes to inclusive economic development.

**Table 18:** Socio-economic impact analysis: Employment creation and skills transition

Employment category	Construction phase (FTE)	Operation phase (FTE/year)	Skill level	Female participation (%)	Training duration (months)
Electrolyzer manufacturing	312	45	high	28.5	12–18
System installation	425	25	medium	15.2	6–9
O&M technicians	85	180	Medium	22.4	9–12
Control system engineers	68	42	high	31.8	18–24
Project management	112	38	high	42.3	–
Administrative support	95	35	low	68.5	1–3
Supply chain and logistics	150	20	medium	35.6	3–6
Total direct employment	1,247	385	–	29.8	–
Indirect employment (multiplier: 1.75)	2,182	674	mixed	34.2	–
Total employment impact	3,429	1,059	–	31.5	–

**Figure 16:**
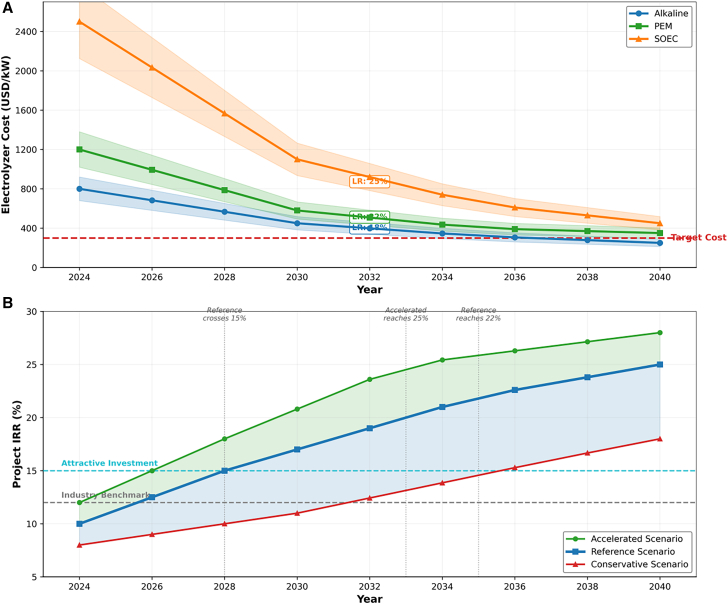
Energy equity analysis: Lorenz curves and Gini coefficients for energy cost burden distribution across income quintiles The Lorenz curves comparing energy cost distributions under baseline and green hydrogen scenarios, with accompanying Gini coefficient calculations quantifying distributional inequality. (A) Electrolyzer cost projections (2024–2040) for Alkaline, PEM, and SOEC technologies with target cost trajectory and 95% confidence intervals. (B) Project IRR evolution under Accelerated, Reference, and Conservative scenarios, with attractive investment (15%) and industry benchmark (12%) thresholds. Note: Gini coefficients calculated from household survey data (*n* = 2,400); error bars represent 95% confidence intervals.

Energy affordability and distributional equity are additional considerations in evaluating the societal impacts of hydrogen energy systems. The energy equity analysis presented in Figure 16 and Table 19 indicates that green hydrogen deployment can reduce household energy burdens across all income groups. Importantly, the relative benefits appear larger for lower-income households, suggesting that renewable hydrogen systems may contribute to reducing energy inequality when implemented through community-level energy systems. This progressive distributional effect arises from increased renewable energy penetration and reduced dependence on fossil fuel price volatility.

**Table 19:** Energy affordability and equity impact across income quintiles

Income quintile	Baseline energy burden (%)	Green H_2_ scenario burden (%)	Burden change (%)	Households in energy poverty (%)	Poverty reduction (%)
Q1 (Lowest 20%)	14.8 ± 1.2	12.3 ± 1.0	−16.9	68.5 → 52.3	23.6
Q2	9.2 ± 0.8	7.8 ± 0.7	−15.2	42.1 → 28.7	31.8
Q3	6.5 ± 0.5	5.6 ± 0.5	−13.8	12.3 → 6.8	44.7
Q4	4.8 ± 0.4	4.2 ± 0.4	−12.5	2.1 → 0.8	61.9
Q5 (highest 20%)	3.2 ± 0.3	2.9 ± 0.3	−9.4	0.0 → 0.0	N/A
Gini coefficient	0.312	0.278	−10.9	N/A	N/A
Energy poverty rate (overall)	24.80%	17.70%	−28.6	N/A	N/A

Environmental sustainability assessments further highlight the broader benefits and trade-offs associated with hydrogen deployment. The life cycle analysis summarized in Table 20 indicates substantial reductions in greenhouse gas emissions and air pollutants relative to conventional hydrogen production pathways. However, increased land use and critical mineral demand associated with renewable infrastructure and electrochemical technologies introduce new sustainability challenges. These findings emphasize the need for integrated planning strategies that combine renewable deployment, mineral recycling systems, and sustainable land use management.

**Table 20:** Life cycle environmental impact assessment (per kg H_2_ delivered)

Impact category	Unit	Green H_2_ (this study)	Gray H_2_ (SMR)	Blue H_2_ (SMR + CCS)	Green vs. gray reduction (%)
Global warming potential	kg CO_2_-eq	2.1 ± 0.4	11.5 ± 1.2	4.8 ± 0.6	81.7
NOx emissions	g NOx	8.5 ± 1.2	28.6 ± 3.5	22.4 ± 2.8	70.3
SOx emissions	g SOx	3.2 ± 0.5	18.7 ± 2.3	15.2 ± 1.9	82.9
Particulate matter (PM2.5)	g PM2.5	1.8 ± 0.3	6.5 ± 0.8	5.1 ± 0.7	72.3
Water consumption	L H_2_O	18.5 ± 2.5	25.8 ± 3.2	32.4 ± 4.1	28.3
Land use	m^2^·year	0.45 ± 0.08	0.12 ± 0.02	0.15 ± 0.03	−275
Mineral resource depletion	kg Sb-eq	0.028 ± 0.005	0.008 ± 0.002	0.012 ± 0.003	−250
Cumulative energy demand	MJ	185 ± 22	178 ± 18	195 ± 24	−3.9

The findings of this study also carry important implications for policy design and industrial development. From a policy perspective, establishing a stable carbon price corridor—potentially within the range of 40–60 USD/tCO_2_—could provide consistent market signals for hydrogen investment while limiting excessive price volatility. Gradual transitions from capital subsidies to performance-based incentives may further encourage efficient long-term system operation. Additionally, regulatory frameworks for microgrid interconnection and ancillary service participation should explicitly recognize the flexibility value provided by hydrogen storage systems.

From an industrial perspective, hydrogen project developers should incorporate uncertainty management into the investment evaluation frameworks. Economic entropy indicators provide a useful metric for capturing the multi-dimensional risk structure of hydrogen supply chains and can complement conventional financial indicators in project assessment. Equipment manufacturers and system operators may also benefit from anticipating market developments under different carbon pricing scenarios and technological learning trajectories. For financial institutions, incorporating uncertainty-aware metrics, such as economic entropy, into project evaluation models may improve risk pricing and investment decision-making.

Finally, the broader transition toward hydrogen-based energy systems must be accompanied by proactive strategies addressing workforce transitions and regional economic restructuring. Policies supporting vocational training, industrial diversification, and community energy participation can help ensure that the benefits of hydrogen deployment are widely shared. Integrating socio-economic considerations with techno-economic system optimization will, therefore, be essential for achieving a sustainable and equitable hydrogen transition.

### Limitations of the study

This study is subject to several limitations. First, the multi-agent framework relies on Markovian rational-agent assumptions; while strategic behavior scenarios are analyzed in STAR Methods, bounded rationality and cognitive biases among real-world stakeholders may deviate from the modeled behavior. Second, the long-term technology roadmap depends on projected learning curve parameters, rather than realized data; actual cost trajectories may diverge due to supply chain disruptions or policy discontinuities. Third, empirical validation is geographically concentrated in the Yangtze River Delta; generalizability to regions with different electricity market designs, carbon pricing mechanisms, or renewable resource profiles requires further testing. Fourth, the five community archetypes represent a limited sample; additional configurations such as remote rural communities or high-latitude locations are not covered. Fifth, the ten-year historical data may not fully capture low-probability extreme events or structural market breaks. Sixth, the life cycle assessment identifies land use and mineral resource trade-offs that are not internalized in the economic optimization. Seventh, the cold-start performance of the adaptive mechanism in new environments with limited data has not been systematically quantified. Finally, the differential privacy frameworks may introduce systematic biases in resource allocation through constraint enforcement projection that require further characterization.

## Resource availability

### Lead contact

Requests for further information and resources should be directed to and will be fulfilled by the lead contact, Chunzhong Li (120120038@aufe.edu.cn).

### Materials availability

This study did not generate new unique reagents.

### Data and code availability

• Data reported in this paper will be shared by the lead contact upon request.
• This paper does not report original code.
• Any additional information required to reanalyze the data reported in this paper is available from the lead contact upon request.

## Acknowledgments

This work was supported by Anhui Provincial Social Science Innovation and Development Research Project: “Reshaping the Competitiveness of Resource-Based Cities in Anhui Province from the Perspective of Innovation Drive”(2024CX069), Anhui Higher Educational Project of Excellent Scientiffc Research and Innovation Team (2023AH010026); 10.13039/501100001809NSFC (61876203); and Provincial Scientific Research Project (2023AH010008, 2022AH050608, KJ2021A0486, 2023xjzlts031, ACKYA22001, and acwzy2024004).

## Author contributions

Y.C. wrote the main manuscript and conceived the study; C.L. performed the formal analysis and methodology validation; J.W. prepared the figures; G.L. and J.W. supervised the study; C.L. acquired the funding; G.L., Y.C., and J.W. contributed to manuscript review and editing; J.W. managed the project. All authors reviewed the manuscript and have read and approved the submitted version.

## Declaration of interests

The authors declare no competing interests.

## STAR★Methods

### Key resources table

**Table:** 

REAGENT or RESOURCE	SOURCE	IDENTIFIER
**Software and algorithms**		

Python 3.9	Python Software Foundation	RRID:SCR_008394
TensorFlow 2.x	Google	RRID:SCR_016345
Gurobi 10.0	Gurobi Optimization	RRID:SCR_018560
SimPy	SimPy Development Team	https://simpy.readthedocs.io
Soft Actor-Critic	Haarnoja et al., 2018	DOI: https://doi.org/10.48550/arXiv.1801.01290
NSGA-III	Deb and Jain, 2014	DOI: https://doi.org/10.1109/TEVC.2013.2281535

**Other**		

National Carbon Market Data	China ETS	Public registry
Renewable and Load Data	Regional Grid Dispatch	Available upon request

### Method details

#### Community energy system configuration

The study investigates a community-scale integrated energy system that couples renewable electricity generation, hydrogen production, and carbon market interactions within a local microgrid environment. Five representative community archetypes were modeled to capture diverse operational contexts: dense urban, suburban mixed-use, rural township, island community, and industrial retrofit systems.

Each community energy system consists of renewable generation units (photovoltaic and wind), electrolyzers for hydrogen production, hydrogen storage tanks, fuel cells for power reconversion, battery energy storage systems, and a local microgrid infrastructure enabling coordinated energy dispatch.

The primary reference community includes 480 residential households together with mixed commercial and public facilities. Installed capacities include 2 MW photovoltaic generation, 1 MW wind generation, a 500 kW alkaline electrolyzer, 1000 kg hydrogen storage capacity, a 300 kW fuel cell system, and a 500 kWh lithium iron phosphate battery. System operations are simulated at a temporal resolution of 15 min.

#### Data sources and temporal coverage

Operational data cover the period from January 2015 to December 2024. Renewable generation data were derived from regional meteorological measurements and historical dispatch records. Electricity demand profiles were obtained from smart meter datasets recorded at 15-min intervals. Carbon price data were obtained from the national carbon emissions trading system registry.

To ensure model robustness, long-term historical data were used to capture seasonal variations in renewable generation, load patterns, and carbon market fluctuations.

#### Uncertainty characterization

Multiple sources of operational uncertainty were incorporated into the simulation framework. Measurement uncertainty in power monitoring systems was assumed to be ±2%, renewable generation forecasting uncertainty ±3%, and carbon price forecasting uncertainty ±8%.

Uncertainty propagation was implemented using Monte Carlo simulation with 1,000 realizations. Each realization generated a stochastic trajectory of renewable output, electricity demand, and carbon price dynamics, enabling probabilistic evaluation of system performance under uncertain operating conditions.

#### Economic entropy modeling

The green hydrogen supply chain was decomposed into four functional segments: hydrogen production, storage and transportation, distribution and consumption, and carbon trading. For each segment, probability distributions describing operational variability were constructed and Shannon entropy was used to quantify the associated economic uncertainty.

Segment-level entropy values were aggregated into total system economic entropy using weighted summation. Weights were determined using a hybrid entropy-weight and CRITIC (Criteria Importance Through Intercriteria Correlation) method to capture both variability and inter-indicator correlation structures.

#### Reinforcement learning implementation

Dynamic entropy regulation was formulated as a partially observable Markov decision process (POMDP). The system state vector includes renewable generation forecasts, electricity demand levels, hydrogen storage states, and carbon market prices. The action space includes electrolyzer power adjustment, storage dispatch decisions, carbon trading volume, and power allocation between energy carriers.

Policy learning was implemented using the Soft Actor–Critic (SAC) reinforcement learning algorithm. The neural network architecture consists of two hidden layers containing 256 and 128 neurons respectively, with ReLU activation functions.

Key training hyperparameters include a learning rate of 3 × 10^−4^, a discount factor (γ) of 0.99, and an automatically tuned entropy temperature parameter. The replay buffer size was set to 10^5^ and the mini-batch size to 256. Training was conducted for 500 episodes using ten independent random seeds to ensure robustness of the learned policy.

#### Carbon–hydrogen–microgrid coupling model

The integrated energy system operates under a coupled electricity–hydrogen–carbon market framework. Electricity market clearing is formulated as a cost-minimization problem subject to power balance and generation constraints. Carbon market equilibrium is modeled through supply–demand matching under government allowance regulations.

Hydrogen pricing is determined based on marginal production cost, storage cost, transportation cost, and market demand conditions. Multi-timescale interactions between markets and system operation are handled through hierarchical temporal abstraction. Reinforcement learning decision layers operate at hourly intervals, while lower-level control layers enforce real-time operational constraints.

#### Multi-agent optimization

Distributed optimization across system components is implemented using the Alternating Direction Method of Multipliers (ADMM). Local decision variables associated with generation units, storage devices, and hydrogen infrastructure are coordinated through global consensus variables and dual updates.

To protect operational data privacy among decentralized agents, differential privacy mechanisms were incorporated. Laplace noise calibrated to sensitivity bounds was applied to exchanged variables. The privacy budget parameter was set to ε = 0.5 using advanced composition accounting.

#### Multi-objective optimization

System planning and operational decisions were evaluated using a four-objective optimization framework. The objectives include minimizing total system cost and economic entropy while maximizing renewable energy consumption rate and carbon emission reduction.

The NSGA-III evolutionary algorithm was applied with a population size of 200 and 500 generations. Simulated binary crossover and polynomial mutation operators were used for solution evolution. Pareto-optimal solutions were evaluated using fuzzy satisfaction functions to identify compromise operating strategies.

#### Scenario generation and reduction

Uncertainty scenarios were generated using Latin hypercube sampling to efficiently explore the multidimensional uncertainty space. A Wasserstein distance–based forward selection algorithm was applied to reduce 10,000 Monte Carlo scenarios to 50 representative scenarios.

Operational decisions were optimized using a rolling-horizon scheduling framework with a 24-h planning window, enabling dynamic adjustment of hydrogen production, storage dispatch, and carbon trading strategies.

#### Economic entropy modeling

The green hydrogen supply chain was decomposed into four segments: hydrogen production, storage and transportation, distribution and consumption, and carbon trading. Segment-level entropy was computed using Shannon entropy formulations based on discretized probability distributions.

Total system economic entropy was calculated as a weighted aggregation of segment-level entropies. Weights were determined using a combined entropy weight and CRITIC method.

#### Reinforcement learning implementation

Dynamic entropy regulation was formulated as a partially observable Markov decision process (POMDP). The state space included renewable output forecasts, demand levels, carbon prices, and storage states. The action space included electrolyzer power adjustment, storage dispatch, carbon trading volume, and power allocation decisions.

Policy optimization was implemented using the Soft Actor-Critic (SAC) algorithm. Neural network architecture consisted of two hidden layers with 256 and 128 neurons using ReLU activation functions. Key hyperparameters included.

• Learning rate: 3 × 10^−4^
• Discount factor (γ): 0.99
• Entropy temperature: automatically tuned
• Replay buffer size: 10^5^
• Batch size: 256

Training was conducted for 500 episodes using 10 independent random seeds.

#### Carbon-hydrogen-microgrid coupling model

Electricity market clearing minimized total generation cost subject to power balance and operational constraints. Carbon market equilibrium was modeled through supply-demand matching with government allowance constraints. Hydrogen pricing incorporated marginal production cost, transportation cost, and demand-supply imbalance adjustment.

Multi-timescale interactions were handled using hierarchical temporal abstraction, where reinforcement learning decisions operated on hourly intervals and physical control layers enforced real-time constraints.

#### Multi-agent optimization

Distributed optimization was implemented using the Alternating Direction Method of Multipliers (ADMM). Local decision variables were coordinated via global consensus variables and dual updates. Differential privacy was incorporated using Laplace noise calibrated to sensitivity bounds.

The privacy budget parameter was set to ε = 0.5 under advanced composition accounting.

#### Multi-objective optimization

A four-objective optimization framework was implemented to minimize economic entropy and total cost while maximizing renewable consumption rate and carbon emission reduction. NSGA-III was applied with.

• Population size: 200
• Generations: 500
• Simulated binary crossover
• Polynomial mutation

Pareto solutions were evaluated using fuzzy satisfaction functions.

#### Scenario generation and reduction

Uncertainty scenarios were generated using Latin hypercube sampling. Scenario reduction was performed using a Wasserstein distance–based forward selection algorithm, reducing 10,000 Monte Carlo samples to 50 representative scenarios.

Rolling-horizon optimization was implemented with a 24-h scheduling window.

### Quantification and statistical analysis

All quantitative results are reported as mean ± standard deviation (SD) unless otherwise specified. The sample size (n) represents independent Monte Carlo realizations, cross-validation folds, or independent training seeds as stated in each analysis.

Statistical analyses were conducted using SciPy (v1.9.0) and Statsmodels (v0.13.0) in Python. Parametric comparisons employed paired or independent t-tests where normality assumptions were satisfied. Multi-group comparisons used one-way ANOVA followed by Tukey HSD post hoc tests. Non-parametric tests were applied when distributional assumptions were not met.

Multiple comparisons were corrected using Benjamini-Hochberg false discovery rate or Bonferroni adjustment as specified in figure legends. Effect sizes were reported using Cohen’s d. Confidence intervals were computed using percentile bootstrap or normal approximation methods.

Significance thresholds were defined as.

• p < 0.05
• p < 0.01
• p < 0.001

Exact *n* values, statistical tests, and definitions of error bars are indicated in figure legends.

#### Data and software availability

##### Software

Custom simulation scripts and reinforcement learning implementation code were developed in Python 3.9 using TensorFlow 2.x. Optimization routines were implemented using Gurobi 10.0. Code is available from the Lead Contact upon reasonable request.

##### Data resources

Renewable generation and load datasets were obtained from regional grid dispatch systems and meteorological records under institutional data-sharing agreements. Carbon price data were obtained from the national carbon emissions trading registry. Processed datasets supporting this study are available from the Lead Contact (120120038@aufe.edu.cn) upon reasonable request.

### Additional resources

No additional external resources were generated in this study.
